# Does context matter for the relationship between deprivation and all-cause mortality? The West vs. the rest of Scotland

**DOI:** 10.1186/1476-072X-10-33

**Published:** 2011-05-12

**Authors:** Sanjeev Sridharan, Julia Koschinsky, Jeremy J Walker

**Affiliations:** 1 The Evaluation Centre for Complex Health Interventions, Keenan Research Centre in the Li Ka Shing Knowledge Institute of St. Michael's Hospital and University of Toronto, Ontario, Canada; 2 School of Geographical Sciences and Urban Planning, GeoDa Center for Geospatial Analysis and Computation, Arizona State University, Tempe 85287, USA; 3 Centre for Population Health Sciences, The University of Edinburgh, Edinburgh EH8 9AG, UK

## Abstract

**Background:**

A growing body of research emphasizes the importance of contextual factors on health outcomes. Using postcode sector data for Scotland (UK), this study tests the hypothesis of spatial heterogeneity in the relationship between area-level deprivation and mortality to determine if contextual differences in the West vs. the rest of Scotland influence this relationship. Research into health inequalities frequently fails to recognise spatial heterogeneity in the deprivation-health relationship, assuming that global relationships apply uniformly across geographical areas. In this study, exploratory spatial data analysis methods are used to assess local patterns in deprivation and mortality. Spatial regression models are then implemented to examine the relationship between deprivation and mortality more formally.

**Results:**

The initial exploratory spatial data analysis reveals concentrations of high standardized mortality ratios (SMR) and deprivation (hotspots) in the West of Scotland and concentrations of low values (coldspots) for both variables in the rest of the country. The main spatial regression result is that deprivation is the only variable that is highly significantly correlated with all-cause mortality in all models. However, in contrast to the expected spatial heterogeneity in the deprivation-mortality relationship, this relation does not vary between regions in any of the models. This result is robust to a number of specifications, including weighting for population size, controlling for spatial autocorrelation and heteroskedasticity, assuming a non-linear relationship between mortality and socio-economic deprivation, separating the dependent variable into male and female SMRs, and distinguishing between West, North and Southeast regions. The rejection of the hypothesis of spatial heterogeneity in the relationship between socio-economic deprivation and mortality complements prior research on the stability of the deprivation-mortality relationship over time.

**Conclusions:**

The homogeneity we found in the deprivation-mortality relationship across the regions of Scotland and the absence of a contextualized effect of region highlights the importance of taking a broader strategic policy that can combat the toxic impacts of socio-economic deprivation on health. Focusing on a few specific places (e.g. 15% of the poorest areas) to concentrate resources might be a good start but the impact of socio-economic deprivation on mortality is not restricted to a few places. A comprehensive strategy that can be sustained over time might be needed to interrupt the linkages between poverty and mortality.

## Background

The goal of this study is to explore the role of spatial heterogeneity in the relationship between socio-economic deprivation and mortality. There is a growing body of research exploring the contextual relationship between deprivation and mortality. More generally, the last decade witnessed a surge in epidemiologic research emphasizing the context-sensitive nature of the relationship between health outcomes and their determinants. That context matters might seem obvious but has often been neglected in traditional study designs. Traditionally, studies have often modelled health outcomes as a function of individual characteristics, assuming that individuals' behaviour and health outcomes are independent of other individuals and of neighbourhood or regional characteristics [1]. A research focus on multi-level modelling [2], neighbourhood effects [3] and built environment [4,5] begins to address this gap. This body of research focuses on factors such as the interaction between individual level and area-level determinants of health outcomes, on the mediating effect of social interactions and on how urban form is related to health outcomes such as obesity. Although these contextual factors are often implicitly spatial, an explicit focus on spatial heterogeneity is still rare (see [6,7] for exceptions).

### Homogeneity and Heterogeneity in the Deprivation - Mortality Relationship

The relationship between area-level measures of socio-economic deprivation and all-cause mortality has been extensively researched [8-15]. While such research often assumes that the relationship between deprivation and mortality is homogeneous and uniform over space, the presence or absence of heterogeneity in the deprivation-mortality relationship can provide important clues to the mechanisms and contexts through which deprivation can impact mortality [16,17], and inform how to respond to socio-economic deprivation, and how to shape policy aimed at reducing health inequalities. For example, *Delivering for Health*, a key health policy document in Scotland, promotes health interventions in the poorest areas as one approach to reducing health inequalities [18]. A relevant question here is whether areas that have high levels of deprivation *and *strong relationships between deprivation and mortality should be targeted.

While most work on heterogeneity in the relationship between risk factors and health has been at the individual level [19-21], recent research has considered the relationship of deprivation to health (broadly defined) both spatially and across multiple levels [22]. Understanding variation at the area level requires methodological approaches that can model and estimate such heterogeneity, but methods commonly used to model the relationship between deprivation and mortality frequently assume that the relationship is uniform across space [23].

The question addressed in this article is whether the relationship between socio-economic deprivation and mortality is indeed the same irrespective of context. One of the assumptions often made in modelling this relationship is that it will remain the same across space. There is little justification presented in the literature as to why the deprivation-mortality relationship will be homogeneous across space. Although the homogeneity of this relationship over space is an empirical question most of the published literature does not formally test this assumption.

There are competing views in the literature on the homogeneity or heterogeneity of the deprivation-mortality relationship. One recent line of evidence suggests there are some good reasons for the deprivation-mortality relationship to be homogeneous over space. Within this viewpoint, the impact of socio-economic deprivation on mortality is so strong that contextual factors might do little to alter this relationship. There is some evidence on the stability (both temporally and spatially) of the deprivation and mortality relationship. For example, a recent article finds temporal stability in the relationship between socio-economic deprivation and mortality over a hundred years in England and Wales: Gregory concludes, "There was no evidence of a significant change in the strength of the relation between deprivation and mortality between the start and end of the 20th century. Despite all the medical, public health, social, economic, and political changes over the 20th century, patterns of poverty and mortality and the relations between them remain firmly entrenched" [24] p. b3454. Dorling et al. reach a similar conclusion: "Contemporary patterns of some diseases have their roots in the past. The fundamental relation between spatial patterns of social deprivation and spatial patterns of mortality is so robust that a century of change in inner London has failed to disrupt it" [25] p. 1547.

On the other hand, evidence for the possible complexities of mechanisms linking deprivation and mortality can be found in Macintyre et al. [26]. Macintyre finds that some poorer areas can also have greater environmental resources that can moderate the toxic impacts of socio-economic deprivation: "Thus there are understandable contextual reasons for a variety of distributional patterns, and it would be sensible not to assume that environmental resources are more likely to be concentrated in better off areas and unavailable to those in poorer areas" [27] p. 5.

### The Deprivation - Mortality Relationship in Scotland

Contextual factors considered in the literature include urban vs. rural location [28], ethnic groups [29], and country contexts [30]. A promising environment for investigating heterogeneity in the deprivation-mortality relationship is provided by the case of Scotland. All-cause mortality is higher in Scotland than in most other Western European countries of comparable wealth and Scotland's mortality rose relative to that for England and Wales from the 1980s onwards [8,9,11]. A recent assessment of Scotland's mortality experience concluded that the expectation of life for Scottish men and women in 2006 was, respectively, around one year and two years lower than the European Union average [10]. Four Scottish Council areas (out of a total of 32) recorded SMRs in 2006 that were more than 10 percent higher than the Scottish average [10]. All four of these areas are located in West Central Scotland. The area with the 'worst' mortality experience in 2006 - Glasgow City - recorded an SMR that was 26 percent higher than the Scottish average (itself around 14 percent above the UK average) [10]).

Attempts to account for Scotland's poorer mortality experience relative to the rest of the UK have highlighted differences in socio-economic deprivation as a possible explanatory factor [12]. However, at the same time, an analysis of patterns of deprivation between 1981 and 2001 concluded that from 1991 onwards, measures of socio-economic deprivation no longer explained most of the excess mortality observed in Scotland [9].

It may reasonably be asserted that regional differences in Scotland (specifically, a contrast between the West and the remainder of the country) are evident in a number of factors: commercial/industrial, religious/cultural and (possibly) climatic. Consequently, the contexts in which the (unknown) processes shaping any hypothesised relationship between socio-economic deprivation and mortality operate are not uniform. Given this heterogeneity of regional context, it is reasonable to expect that the nature of the observed association between deprivation and mortality may differ between the West and other regions.

When investigating geographical variations in mortality within Scotland (in particular, the poor mortality experience of the West)^i^, regional differences in factors plausibly associated (not necessarily causally) with differential mortality rates must be considered. One such factor is the nature of regional commercial activity, past and present. The city of Glasgow formerly hosted Scotland's greatest concentration of heavy industry, but (in common with the West of the country generally) experienced de-industrialization on a massive scale in the latter half of the 20th century [13]. In contrast, other regions were (and remain) markedly less industrial in character.

Although the data in this study did not allow us to account for religious affiliation, a second distinctive feature of the West relates to a higher prevalence of Catholicism than in most other areas of Scotland. This disparity is potentially relevant to regional mortality because Catholic religion in Scotland mainly indicates Irish ancestry [31,32], and Irish background is associated with disadvantage in health [33] and socio-economic position [34]. Such findings raise the possibility that Scottish inter-regional differences in religious affiliation (especially the 'West versus the rest' contrast) may act as a proxy for variations in other behavioural, cultural or lifestyle factors which potentially relate to observed mortality differentials.

One further factor with possible relevance to regional mortality differences is the influence of local meteorological conditions. One theory with plausible relevance to the Scottish mortality experience involves the 'inverse housing law' identified by Blane et al. [35,36]. This postulates that areas of the UK that experience harsher local climatic conditions also have poorer housing, and parts of Scotland (including the West) are identified as suffering both poor climate and poor housing [35] p. 746, Figure 1. This pattern of association between climate and housing conditions exhibits relationships with both respiratory health [35] and hypertension [36].

**Figure 1:**
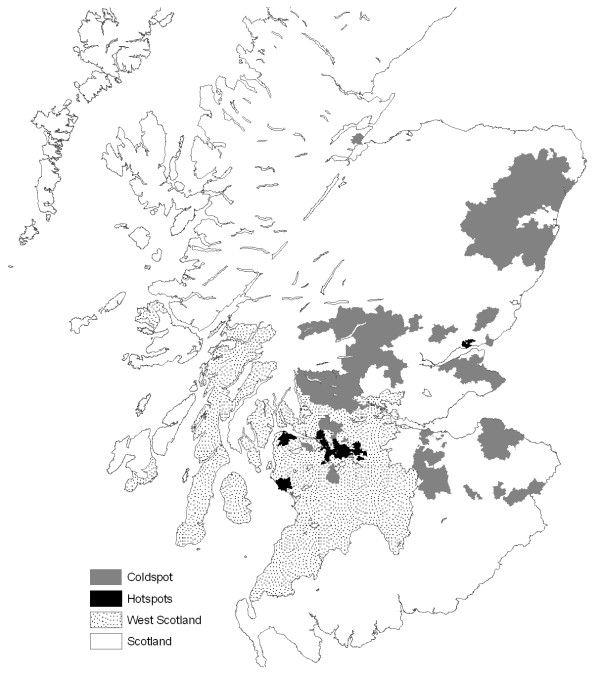
**Map of Deprivation Clusters**. This map illustrates that clusters of high deprivation values are concentrated in the West of Scotland compared to clusters of low deprivation in the rest of Scotland.

### Hypotheses

The hypothesis examined in this article is that the relationship between socio-economic deprivation and mortality differs statistically across the regions of Scotland. Based on the existing evidence presented above, we anticipate the coefficient linking deprivation to mortality in the regression models to be statistically significantly larger in the West than in the other regions of Scotland. The null hypothesis to be tested suggests that, on average, the relationship between deprivation and all-cause mortality remains constant across regions, i.e. is not affected by regional context.

### Data

The data for this study were obtained from Information Services Division (ISD), a subunit of NHS National Services Scotland. Data on all-cause mortality originate from the General Register Office for Scotland while the other measures in the study are from the 2001 census [10]. In this study we focus on the spatial arrangement of communities at the finest geographical scale for which data were accessible in order to avoid some of the 'smoothing' of population characteristics, which may occur when using physically large areal units. However, physically small areal units often contain small residential populations, few deaths and correspondingly unstable mortality rates. Scotland's fragmented landscape also presents challenges for analyses focused on the spatial arrangement of population characteristics; administrative units are often physically split (across islands for example). For this exploratory study we therefore restricted the statistical analyses to postcode sectors with a population of 1,000 or more and to a single physical segment for each sector (e.g. the geographic shapefile contained duplicate values for all separate sub isles of the same island). The original postcode sector map file contained 1,165 records, which included 195 records with populations smaller than 1,000 and 130 records that contained duplicate values or belonged to islands that we excluded from the spatial analysis because they were too removed from the mainland. After excluding these records, we were left with 840 postcode sector records (379 in the West and 461 in the rest of Scotland) to include in the analysis.

Since the question of interest is whether the relationship between deprivation and mortality varies between West Scotland and the other Scottish regions, the two key measures in the article include the 2001 Carstairs score (a composite measure of deprivation) and standardized mortality ratios. These measures are aggregated at the postcode level. The Carstairs score consists of four standardized census variables: adult male unemployment, lack of car ownership, low social class and overcrowding [37,38]. The standardized score for each of the variables is first calculated and then the Carstairs score is computed by summing each of the individual standardized scores. Note that under this method of calculation, the Carstairs score ranges from negative to positive values, which represent a range from very low to very high levels of deprivation.

All-cause mortality by age group and sex is computed as the annual number of deaths within an age group per the population in that group. Mortality ratios for deaths at ages under 75 years are standardized using age and sex specific death rates for Scotland for age groups 0 to 4, 5 to 14, 15 to 24, 25 to 34, 35 to 44, 45 to 54, 55 to 64, and 65 to 74 years. SMRs were based upon deaths registered during a three-year period around the 2001 census and population denominators from the 2001 census. The exclusion of deaths at ages 75 years and over focuses the analysis on premature mortality. Several studies have found premature mortality to be more closely associated with area deprivation than deaths at older ages [12] although this finding is disputed [39]. Hanlon et al's [9] analysis also suggests that the proportion of excess deaths in Scotland in comparison to England and Wales are relatively lower at age 75 years and over than at younger ages. The exclusion of deaths at ages 75 years and over will also reduce the influence that the presence of nursing homes and other institutions with higher concentrations of older persons may have on the death counts within small areas [40]. Data on rainfall (in inches) and temperature (in degrees Celsius) were aggregated at the postcode sector level [41]. Additional file 1: Table S1 summarizes the data.

## Methods

### Spatial Approaches to Studying Area Level Deprivation and Mortality

One promising approach to elucidating the determinants of the seemingly anomalous mortality profile of the West of Scotland involves the adoption of spatial data analysis [15,42,43]. Local small-area variation in mortality lends itself readily to investigation via spatial analysis, the functions of which include detecting spatial patterns in data and formulating hypotheses based on the geography of the data [43]. In this context, the methodology applied in this article is designed to tease out the spatial dimensions of the relationship between socio-economic deprivation and all-cause mortality in Scotland, with a focus on regional differences in this relationship and spatial clustering of mortality rates. To do so, we start with exploratory spatial data analysis (ESDA) and descriptive statistics for an overview of the spatial distribution of mortality and deprivation, the extent of local and overall clustering of these values, and their bivariate correlation. This exploratory stage is formalized in a diagnostic test of spatial dependence of the Ordinary Least Squares (OLS) residuals to detect potential patterns of spatially correlated values (in either mortality rates or in the error term) in a particular postal code sector and neighbouring sectors. Next we estimate a spatial regression model of the mortality-deprivation relationship, controlling for mortality rates at neighbouring locations as well as standard covariates. To test for spatial heterogeneity in the deprivation-mortality relationship, this model is extended to include so-called spatial regimes, the West and the rest of Scotland.

The purpose of the exploratory spatial data analysis is 1) to better understand the extent to which the relationship between mortality and deprivation is consistent between the West and other regions of Scotland (spatial heterogeneity), and 2) to assess the extent of clustering of mortality rates or deprivation between a postcode sector and its neighbours and to identify where these clusters are located (spatial autocorrelation). Global and local Moran's I are used as the statistical tests to identify the extent of overall clustering and the location of the local clusters [42]. The exploratory analysis conducted in this article is limited to univariate or bivariate relationships (e.g. analyzing the relationship of the same variable in different locations). Statistically significant hotspots and coldspots are identified relative to the mean (e.g., above-the-mean SMR values in a given postcode sector and its neighbours). These ESDA methods are implemented through OpenGeoDa [44].

To extend this analysis to multivariate regression modelling, we first test whether spatial autocorrelation needs to be accounted for. A diagnostic test is used to determine whether spatial autocorrelation is present in the OLS residuals. The null hypothesis here is that of spatial randomness, in other words, that SMR values in a given postcode are not related to those in neighbouring postcodes. This article utilizes Lagrange Multiplier (LM) tests for spatial dependence [45] that point to either a lag or error model alternative as a better fit for the data if the OLS residuals are found to be spatially autocorrelated [46]. As discussed in the results section, the LM Lag test was significant for the data used in this article, pointing to a spatial lag model as the best fit for the data.

The spatial lag model specification adds the average of neighbouring values of the dependent variable as a predictor to the model. In other words, SMRs are not only modelled as a function of covariates in the same postcode sector (such as sex or temperature) but also of SMRs in neighbouring postcode sectors. If the spatial lag (i.e. the average neighbouring SMR values) is significant, this can arise for different reasons. For example, correlation between SMRs in a given postcode sectors and its neighbours can reflect a process of contagion where neighbours influence a given postcode sector and vice versa. Alternatively, this correlation can be due to spatial measurement error where, for example, the spatial extent of a postcode (which represents an administrative unit for mail delivery purposes) does not correspond well to the spatial extent of the processes that are related to mortality rates. In the first case, values for neighbouring postcode sectors can be related because they influence each other. In the latter, they are correlated due to a mismatch in spatial scale.

Because the spatial lag term is correlated with the error term the spatial lag model needs to be estimated through specialized spatial methods (the use of OLS to estimate this model would generate biased and inefficient coefficients). In this article, we estimate this model through spatial two-stage least squares, which uses the first order of the spatially lagged independent variables (WX) as instruments to estimate the coefficient for the spatially lagged dependent variable, Wy (for details, see [46,47]). One of the other estimation problems that needs to be addressed is related to the variation in population size between postcode sectors: Population sizes range from 1,000 to 20,512 with a mean size of 5,949 persons per postcode sector (and a standard deviation of 2,994 persons; postcode sectors with populations smaller than 1,000 were excluded from the analysis). This variation is problematic because it biases the parameter estimates and is related to variance instability in mortality rates across postcodes, i.e. it means that the mortality rates are associated with varying degrees of precision. To address this problem, we initially weight the linear regression for population size (WLS models 1 and 2 in Additional file 2: Table S2). Since the software tools available to the authors to estimate the spatial lag model do not estimate a spatial WLS model, we compare the results of the WLS model to those of an OLS model with standard errors that are robust to heteroskedasticity (White-adjusted). The beta estimates for the West and rest of Scotland WLS and robust OLS models are very similar for the main variable of interest (deprivation). We therefore then proceed with an estimation of the spatial lag models that are not weighted by population size but corrected for heteroskedasticity.

To incorporate a test of spatial heterogeneity in the association between socio-economic deprivation and mortality, the Scotland-wide model specifications are extended to include so-called spatial regimes [48]. The spatial regimes model allows the covariates and the residual covariance to vary across regions (West vs. Rest of Scotland). In essence, separate coefficients are estimated for the two regimes: West Scotland and the rest of Scotland (SE-North Scotland). This is similar to estimating a separate model for each region with two important differences: 1) the spatial regime approach estimates the standard errors within each regime based on the whole dataset, which results in more precise standard error estimates, and 2) a spatial chow test [48] evaluates whether there is a statistical difference between the coefficients in each regime.

The spatial Chow test is a spatial variant of the Chow test [48] to assess if the null hypothesis of spatial stationarity holds against the alternative of spatial heterogeneity. Specifically, it tests if the coefficients for the same variable remain constant across regions or not and if there is a statistical difference between regions for the model overall. Additional file 2: Table S2 reports the Spatial Chow value and significance level for the model overall at the bottom of the results for each model. The values and significance levels associated with the Spatial Chow test for the stability of individual coefficients across regions is reported as a separate column for each model in this table.

The spatial regimes model provides important clues to the (unobserved) mechanisms by which socio-economic deprivation is connected to mortality. Note that the absence of heterogeneity is in itself indicative of the mechanism that connects deprivation to mortality. The following notation is used for a linear spatial lag model with spatial regimes [46]:

where y is the vector of observations on the dependent variable, Wy the spatial lag term, X is the matrix of exogenous variables, β is the vector of regression parameters (*ρ *is the spatial parameter of Wy, which is estimated for the model as a whole), and *ε *is the vector of regression disturbances (i.i.d). The asterisk indicates that each parameter contains subgroups of observations associated with the two regimes. To illustrate, Model 4 in Additional file 2: Table S2 contains SMRs for 840 postcodes, 13 regressors, and two regimes (West and Rest). In the West of Scotland regime, the regressors will have nonzero values for this regime and zero values for the Rest regime. Conversely, the regressors for the Rest regime contain non-zero values for the Rest regime and zero values for the West regime.

The model of the deprivation-mortality relationship in the two Scottish regions includes a series of control variables that are expected to affect mortality rates. The base spatial regime model specification used in this article is shown in the following equation:

where the dependent variable, SMR, is modelled as a linear function of an intercept, deprivation (CAR), a spatial lag term *Wy *(the average of neighbouring SMR values), a matrix of AGE variables (percentage of population in age groups 0-4, 5-14, 15-24, 25-34, 35-54, 55-64, 65-74), the percentage of males in the population (MALE), an indicator for each of the Glasgow, Edinburgh, Aberdeen and Dundee urban areas (URBAN), mean temperature in degrees Celsius (TEMP), and annual rainfall in inches (RAIN). The four urban indicators are coded as 1 if the postcode sector falls inside the urban areas of Glasgow, Edinburgh, Aberdeen or Dundee, respectively, and zero otherwise. All variables are associated with their respective beta coefficient (in the case of *Wy *, *ρ *is used) whereas alpha represents the coefficient of the constant and *ε *is the error term.

The asterisk indicates that the model is estimated for two regimes: West and Rest (Southeast-North).^ii ^These regimes are based on the classification of the three Health Boards (West, Southeast, and North) used by the Cancer Team of Information Services Division (ISD) Scotland.^iii ^The West region consists of Ayrshire & Arran, Argyll & Clyde^iv^, Forth Valley, Glasgow, Lanarkshire. The North region includes Grampian, Highland, Orkney, Shetland, Tayside, Western Isles; and the Southeast region contains Borders, Fife, Lothian, Dumfries & Galloway. The North and the Southeast region were consolidated to create the "SE-North Scotland category" (also abbreviated as "Rest" in comparison to "West"). Note that the coefficient for the spatial lag term is estimated for the model as a whole (as opposed to each region) in the regimes specification.

To test the robustness of the results to different model specifications, we compare results that are weighted for population size (Model 1 and 2),^v ^control for spatial autocorrelation (Models 5-10) and heteroskedasticity (all models), assume a non-linear relationship between mortality and deprivation (Model 7-10), break the dependent variable into male and female SMRs (Models 8-9), and distinguish between West, North and Southeast regions (Model 10) in Additional file 2: Table S2. Additional file 2: Table S2 presents the Spatial Chow test values and significance levels for all spatial regime models in two versions: As a test of the differences between regimes for the overall model (bottom of Additional file 2: Table S2) and as a test for the stability of individual coefficients across regimes (last column for each model). For Model 1 (OLS), the R^2 ^is reported. For the other spatial lag models, a pseudo R^2 ^value is used (pseudo R^2 ^because this ratio of the variance of the predicted values over the variance of the observed values for Y is not equivalent to R^2^).

Although these methods account for many dimensions of the deprivation-mortality relationship, several limitations remain. One of them is related to the exclusion of postcode sectors with populations below 1,000 persons. This exclusion creates "spatial gaps" in the map of postcode sectors. To address this gap we used Thiessen polygons to obtain a contiguous area of postcode sectors (see endnote 8). The disadvantage of this approach is that areas become neighbours that are not actually geographical neighbours (although there might be more exchange between larger-size areas through transportation than between rural and urban areas). Also, because this study excludes postcode sectors with smaller populations, the findings do not apply to more rural areas. Another limitation is the abovementioned assumption that the spatial regression results would not significantly differ if weighted for population.

## Results

In summary, the exploratory spatial data analysis results revealed SMR and deprivation hotspots in the West and coldspots in the rest of Scotland. However, the spatial regression results suggest that the relationship between all-cause mortality and socio-economic deprivation is rather constant in both regions. This result is robust to a number of specifications, including weighting for population size, controlling for spatial autocorrelation and heteroskedasticity, assuming a non-linear relationship between mortality and deprivation, breaking the dependent variable into male and female SMRs, and distinguishing between West, North and Southeast regions.

### Exploratory Spatial Data Analysis

To summarize the results, the initial exploratory spatial data analysis reveals concentrations of high SMR and deprivation values (hotspots) in the West of Scotland and concentrations of low values (coldspots) for both variables in the rest of the country. The question is whether this spatial heterogeneity in the distribution of SMR and deprivation values in the two regions is associated with a different relationship between mortality and deprivation (in terms of the intercept or slope of their respective coefficients). It turns out the answer is no - we cannot reject the null hypothesis of spatial homogeneity in the deprivation-mortality relation in Scotland for the data analysed in this article. As the spatial modelling results below will demonstrate, the relationship between socio-economic deprivation and mortality remains essentially constant in the West versus the rest of Scotland, despite the contextual differences that characterize the two regions. This result also holds when the regions are separated into West, Southeast and North, and for male and female SMRs as the dependent variables.

As described in Additional file 1: Table S1, the SMR for the overall population, as well as the SMRs for males and females, are considerably higher in the West as compared to the rest of Scotland. Similarly, the levels of socio-economic deprivation are also considerably higher in the West as compared to the rest of Scotland. Note that the pattern of results in Additional file 1: Table S1 provides few clues regarding heterogeneity in the relationship between socio-economic deprivation and mortality.

**Figure 2:**
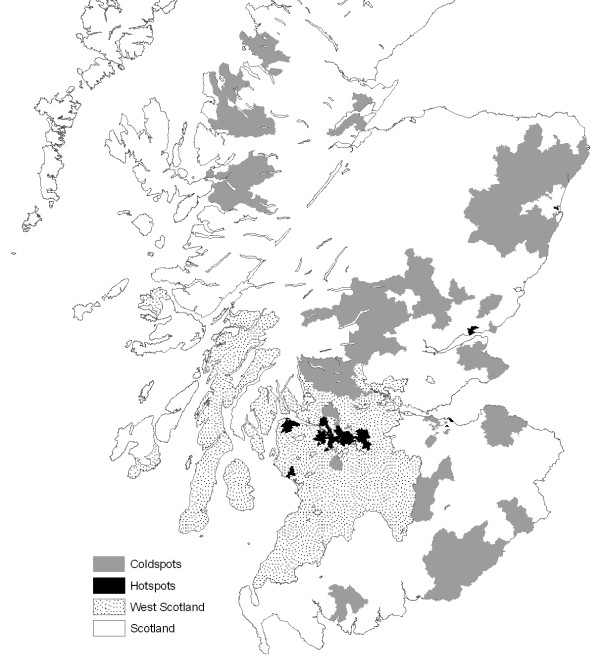
**Map of SMR Clusters**. Similar to the pattern in Figure 1, this map illustrates that clusters of high SMRs values are concentrated in the West of Scotland compared to clusters of low SMRs in the rest of Scotland.

To get a better sense of the spatial distribution of SMR and deprivation, Figures 1 and 2 present maps of local indicators of spatial association (local Moran's I, as mentioned above) for SMR and deprivation in West Scotland and the remaining regions. Two types of spatial association are highlighted: clusters of high values (hotspots) and clusters of low values (coldspots).^vi^

**Figure 3:**
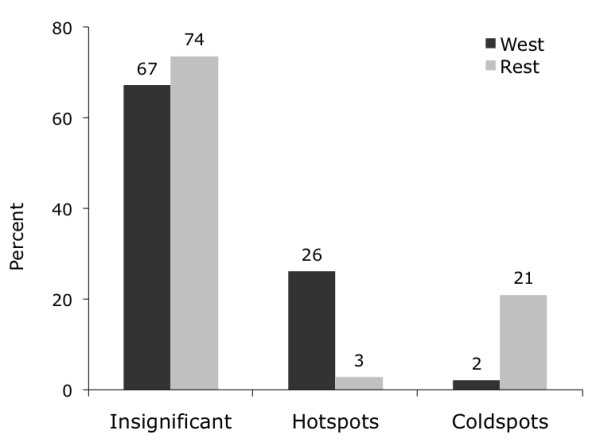
**Percentage of SMR Hotspots and Coldspots**. 26% of all-cause mortality hotspots are in the West of Scotland compared to 21% of coldspots in the rest of Scotland.

**Figure 4:**
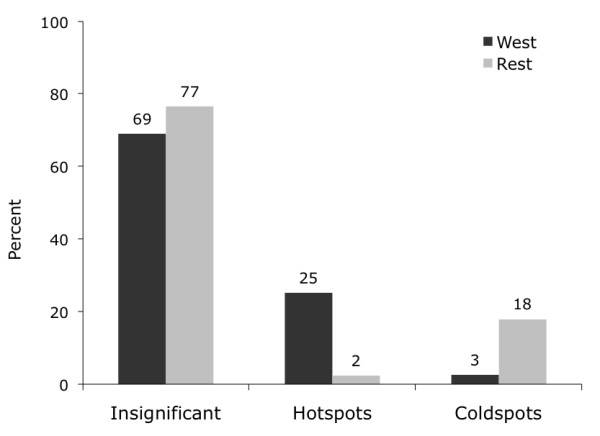
**Percentage of Deprivation Hotspots and Coldspots**. 25% of deprivation hotspots are in the West of Scotland compared to 18% of coldspots in the rest of Scotland.

**Figure 5:**
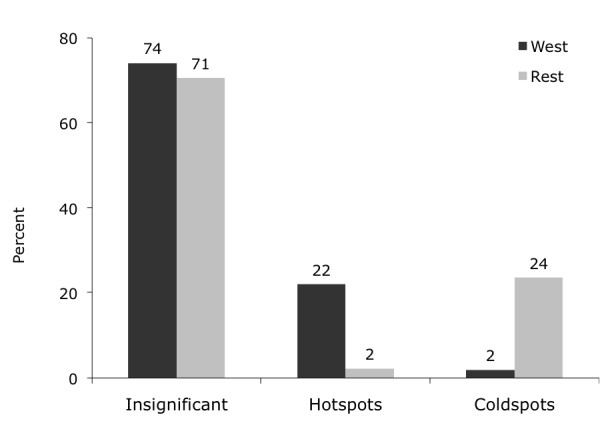
**Percentage of SMR-Deprivation Hotspots and Coldspots**. 22% of all-cause mortality with neighbouring deprivation hotspots are in the West of Scotland compared to 24% of coldspots in the rest of Scotland.

What this analysis demonstrates is that hotspots of both all-cause mortality and deprivation are concentrated in West Scotland while coldspots of both are primarily found in the remaining regions. This finding is quantified in Figures 3, 4 and 5, which --for the West vs. the rest of Scotland-- compare the proportion of local indicators of spatial association (LISA) cluster cores for SMR clusters (Figure 3), deprivation clusters (Figure 4) and clusters of two separate variables: SMRs in a given postcode with the average deprivation index of its neighbours (Figure 5). The latter examines the bivariate spatial relationship between deprivation and the neighbouring values of mortality for both Western and other regions.

While the proportion of insignificant (i.e. non-clustered) postcodes is comparable between the West and Rest of Scotland in all three cluster cases, the proportion of hotspots and coldspots reverses in all three cases. Clusters of high SMRs and deprivation are concentrated in West Scotland: about a quarter of all postcodes in this area constitutes a hotspot. In contrast, this is only true for 2-3 percent of postcodes in the Rest of Scotland. The pattern of coldspots mirrors this finding: low values of SMRs and deprivation are clustered in the rest of Scotland at 18-21%, while such coldspots are only found in 2% of the cases in West Scotland. The same pattern is true for clusters of SMRs and neighbouring deprivation.^vii^

In Scotland, all-cause mortality and socio-economic deprivation measures in a given postcode are significantly clustered with those in neighbouring postcodes (Moran's I = 0.46, which is highly significant). This can include clustering of low and/or high values. When West Scotland is excluded, Moran's I is 0.29 for SMRs and 0.32 for Carstairs (both still highly significant). However, what this suggests is that both variables are more strongly spatially clustered throughout West Scotland compared to the rest of Scotland.

Moving beyond a univariate analysis to bivariate relationships, the (non-spatial) Pearson correlation for SMR and Carstairs is examined. It turns out to be very similar for the West and Rest regions. Strong associations are observed for both the regions with close to 65% of the variance in SMR explained by Carstairs. For the Western region the correlation coefficient between SMR and Carstairs is 0.83 (p < 0.01); in the remaining region the correlation coefficient is 0.81 (p < 0.01). This similarity is striking in light of the fact that, respectively, high values of mortality and deprivation are clustered in the West of Scotland while low values cluster in the rest of the country. In other words, the fact that the relationship is so similar is surprising if one expected a differential impact of contextual factors on SMRs in the West vs. the rest of Scotland.

### Spatial Modelling

The main result is that socio-economic deprivation is the only variable that is significant at the 0.001 level in all models. However, in contrast to the expected spatial heterogeneity in the deprivation-mortality relationship, this relation does not vary between regions in any of the models (none of the values of the Spatial Chow test for differences in the deprivation coefficients across regions is significant). In other words, the null hypothesis that, on average and *ceteris paribus*, the same relationship between deprivation and mortality holds across regions cannot be rejected. However, it is surprising from a perspective that expected the different contexts of the West and the rest of Scotland (including clusters of higher levels of deprivation and mortality in the West) to be related to differences in the correlations between deprivation and mortality. Our findings support research such as that of Gregory [24] and Dorling et al. [25], which found a rather constant relationship between mortality and deprivation across time.

To determine whether the clusters found in the exploratory stage result in spatially correlated errors, we estimate the model in equation 3 with OLS (without the spatial lag term and the regimes) to obtain the LM test results for spatial autocorrelation in the OLS residuals. The LM-Lag test result (19.09) turned out to be more statistically significant (p-value: 0.000012) than the LM-Error test result (12.84 with p-value of 0.00034). This result indicates that the OLS residuals are spatially autocorrelated in this model and thus it motivates the estimation of the spatial lag specification in Models 5 to 10. Additional file 2: Table S2 summarizes all model results.

The models with total SMRs and one deprivation variable explain a larger proportion of the variability in SMRs (between 74-79%)^viii ^than those with multiple deprivation variables and SMRs by sex where the explained variation drops to 60-64%. The model has a better fit for male than female SMRs: in the case of female SMRs, the explained variation in SMRs is lowest (Model 9: R^2 ^= 50%).

The following discussion analyzes the extent to which this main result is robust to different model specifications. In short, the finding of a lack of spatial heterogeneity holds across the variety of model specification tested in this analysis. Overall, both weighting for population size (WLS) and controlling for spatial correlation of SMR values (spatial lag model) reduces the difference between the deprivation coefficients for the West and rest of Scotland by decreasing the size of the West estimate. Specifically, if deprivation (Carstairs) is included as a single variable, assuming a linear relationship with SMRs, robust OLS estimates the parameter of this relationship to be 9.27 for all of Scotland (Model 3). Adjusting for population size through WLS reduces this parameter estimate to 8.65 (Model 1) while adjusting for the correlation of SMRs between a postcode sector and its neighbours through the spatial lag model further reduces the parameter estimate to 8.33 (Model 5). Although the differences between WLS and OLS estimates are larger for all of Scotland, they are more similar for the models that separate the West vs. the rest of Scotland: for the West, 9.37 (WLS, Model 2) compared to 9.78 (OLS, Model 4) and for the rest, 8.14 (WLS, Model 2) compared to 8.34 (OLS, Model 4).

The spatial model's (Model 6) estimate of the deprivation parameter for the rest of Scotland is similar to that of the WLS and OLS estimates (8.13). However, at 8.86, the beta coefficient for the West is lower than that of WLS and OLS. The spatial models do not adjust for population size, but the fact that the WLS model reduces the size of the West deprivation coefficient could indicate that, if anything, the West vs. Rest mortality-deprivation relationship might be even more similar if the spatial model was weighted for population size. The spatial Chow test for the deprivation-mortality relationship comparing the West and rest of Scotland is not significant for any of the spatial models (Models 6-10, with smaller West estimates). However, even for the model with the largest West vs. Rest difference (the robust OLS Model 4), the spatial Chow test fails to detect a significant difference. In other words, the homogeneity of the deprivation-mortality relationship seems to be robust to these alternative model estimations.

Since there is no reason to assume linearity in the deprivation-mortality relationship, Models 7-10 include separate indicators for whether a postcode is in the bottom or top third of deprivation values.^ix ^As the exploratory analysis showed, there are higher levels of socio-economic deprivation in the West than the rest of Scotland. This means that these indicators have different average values in the two regions: the high deprivation indicator in the West has a mean Carstairs value of 5.49 compared to that of 3.81 in the rest of the country. The low Carstairs means are similar (-1.6 West vs. -1.5 Rest) while the average for the excluded middle value category is higher in the West (-0.10 vs. -0.56 Rest). Hence one should only compare results across models within the respective West and Rest regions and use the spatial Chow tests for the between-region comparison since the spatial Chow test specifically tests for differences in means across regions.

As perhaps expected, the strongly significant deprivation-mortality relationship identified in Models 1-6 (single deprivation variable) seems to be driven by the highest deprivation levels. While most of the low deprivation indicators differ only at the 0.05 significance level from the excluded middle deprivation category, the high deprivation indicator differs significantly at the 0.001 level in all cases but one. In the West, the parameter estimate for high deprivation is 27 (Models 7 and 10), which is higher for males (31) than females (24) - a pattern that holds in the rest of Scotland. However, consistent with the findings in Models 1-6, even when deprivation is measured in discrete categories (Models 7-10), the spatial Chow test indicates that there are no statistically significant differences in the deprivation parameter estimates between regions in low or high deprivation categories in any of these models.

Many of the age variables (except for the youngest age category) are not only significantly related to mortality but also differ in their relationship with mortality between regions in the models with spatial Chow tests. Since 75+ years is the excluded age category, the coefficients for the other age intervals are negative, i.e. associated with comparatively lower SMRs. Postcode sectors in urban areas, those with higher average temperatures and more rainfall are associated with higher SMRs in some of the Scotland-wide models (WLS; in the OLS case this is only true for the two climate variables while for spatial lag model 5 only the temperature variable is significant). However, these relationships lose or weaken in significance in the regional West vs. Rest models. The percentage of males in a postcode is not related to mortality in any model except negatively (-2.79 at a p-value of 0.001) in the Southeast of Scotland (Model 10).

Neighbouring SMRs are a highly significant predictor of SMRs in all of the models (at the 0.001 level) where deprivation is separated into low and high categories (coefficients range between 0.38 and 0.43). They have smaller values in Models 5 and 6 (0.11 with p-value 0.01 and a non-significant 0.07) where deprivation is included as a single variable. This suggests that neighbouring SMRs primarily play a role in models where high deprivation is also strongly related with high mortality rates.

## Discussion

In this article, we asked whether context matters for the relationship between socio-economic deprivation and all-cause mortality in the West vs. the rest of Scotland. We had anticipated finding a heterogeneous relationship between socio-economic deprivation and mortality across the regions of Scotland. To our surprise, the relationship between deprivation and mortality did not differ between the West and the rest of Scotland (especially when postal code sector population size and spatial clustering of mortality rates are taken into account). In other words, even though the levels of socio-economic deprivation and mortality are both higher in West Scotland than the rest of Scotland (as the exploratory analysis showed), the deprivation-mortality relationship is nevertheless comparable in both regions. Within each region, areas with higher deprivation scores also have higher mortality ratios. This result is consistent with the remarkable temporal stability found in Dorling et al. [25] and Gregory: "Even when the effects of modern deprivation are taken into account, mortality patterns from the 1900s still have a significant relation with mortality today and this affects most major modern causes of death" [24] p. b3454. Further, the practical questions that the stability in the relationship between deprivation and mortality raises are what governments and other organizations can do to interrupt this stable relationship between socio-economic deprivation and mortality. More broadly, the question is what this means for solutions to address the linkages between socio-economic deprivation and mortality [24].

The homogeneity in the deprivation-mortality relationship and the absence of a contextualized effect of region points to the very strong impacts of deprivation on mortality. This finding also calls attention to the importance of taking a broader strategic policy that can combat the toxic impacts of socio-economic deprivation on health. Focusing on a few specific places (e.g. 15% of the poorest areas) to concentrate resources might be a good start but the impact of deprivation on mortality is not restricted to a few places. A comprehensive strategy that addresses one of the most powerful social determinants of health - poverty--is needed to improve health. It also calls into question the practice of running short-term interventions: if the linkages between socio-economic deprivation and mortality are so stable, governments need to rethink the strategy of trying short term "feel good" interventions for brief periods. Instead, a focus on interventions that can be sustained over the long haul might be needed to interrupt the linkages between poverty and mortality.

While we found a homogeneous relationship between socio-economic deprivation and mortality across regions in Scotland, one of the interesting implications of the methodological approach implemented in this article has been to start with an expectation of heterogeneity, rather than *a priori *assume a homogeneous relationship between deprivation and mortality.

A focus on heterogeneity or homogeneity of linkages might also have practical consequences for locating interventions in specific places. Exploring the presence of heterogeneity in linkages between socio-economic deprivation and mortality can help inform the location of interventions. For example, an important research question for future research is whether interventions should be located in places where the relationship between socio-economic deprivation and mortality is strongest. Addressing this question can help plan more spatially informed interventions.

## Conclusions

In summary, this study demonstrated a role for spatial analysis methods in illuminating one of the central questions of health inequalities research: the relationship between deprivation and mortality. Although the substantive findings are restricted to Scotland, the study was conceived partly as a methodological illustration of the utility of testing spatial approaches. Such approaches have widespread potential, not only to further elucidate the determinants of mortality (and morbidity) in Scotland, but to investigate a wide range of risk/health associations in many other intra- and international contexts.

This article has explored the application of spatial methods in understanding the heterogeneity in the relationship between deprivation and mortality. As is common in such investigations, the analysis proceeded by assuming a linear relationship between deprivation and mortality and then estimated the mortality-deprivation relationship for low and high categories of deprivation. One area for future investigations is to explore the spatial heterogeneity of deprivation-mortality relationships with more sophisticated non-linear estimates.

This article does not focus on mechanisms to explain the observed homogeneity in the deprivation-mortality relationship. Future investigations need to explore the mechanisms that can explain why no contextual relationships are obtained. A focus on mechanisms can also be aided by considering multiple operational definitions of deprivation and health. For example, an important area for future research is to examine the robustness of the spatial homogeneity of deprivation-mortality relationship to changes in definitions of deprivation. Future research needs to examine multiple measures of health outcomes to determine whether spatial heterogeneity is observed for morbidity and at different units of analysis. Differences in the spatial heterogeneity results might provide clues to the mechanisms by which socio-economic deprivation impacts health.

The focus of the analysis described here was at the post-code sector. Given the Modifiable Area Unit Problem (MAUP), future research needs to explore if homogeneous relationships obtained in this article are also observed at other units of analysis. A focus on the spatial heterogeneity of deprivation-mortality relationships can also help define a broader research agenda--heterogeneity can take many different forms and the focus in this article has been on heterogeneity between spatial units. There is also a need to pay attention to heterogeneity within places, e.g. as argued by Haynes and Gale [49].

In addition to the above methodological challenges, a few other points need to be considered in future research. The results in this paper were obtained by excluding postcodes with population size less than 1,000 - future research needs to explore the stability of the deprivation-mortality relationship with the inclusion of the postcodes with smaller population size. Future research utilizing the Scottish postcodes also needs to explore the implications of multiple physical segments per sector. In short, given the multiple methodological challenges of spatial dependence and variance instability, future explorations of the spatial relationship between deprivation and mortality should consider multiple models, multiple methodologies and focus on multiple units of analysis.

We have used a conceptualization of regime (West and rest of Scotland) that is both substantively driven and also driven by convenience. Other theoretically informed approaches might also be possible - for example, it might be useful to compare the heterogeneity in the linkages between socio-economic deprivation and mortality in urban areas with rural areas, or Glasgow with other cities in Scotland. We have implemented a spatial regimes model to study heterogeneity in linkages. Future research can also implement other methodological approaches that include the use of Geographically Weighted Regression to study the spatial variation of coefficients across space [50,51] or the application of other spatial methods.

## Competing interests

The authors declare that they have no competing interests.

## Authors' contributions

The research design was carried out by SS, JK, and JW. SS and JK made the methodological choices and analyzed the data. The article was written by SS, JK and JW. All authors read and approved the final manuscript.

## Endnotes

^i^Based on the definition of health boards, the West region consists of Ayrshire & Arran, Argyll & Clyde, Forth Valley, Glasgow, Lanarkshire.

^ii^For comparison purposes, Model 7 separates the Rest region into North and Southeast regions.

^iii^For more information, see http://www.isdscotland.org.

^iv^This is based on an older NHS Scotland classification. Argyll and Clyde was a former Health Board of the National Health Service in western Scotland. In April 2006, NHS Scotland dissolved the board and transferred its responsibilities to NHS Highland and NHS Greater Glasgow and Clyde.

^v^Since the spatial lag models can currently only be estimated as robust models without population size adjustments by the authors, we use robust ordinary least squares to compare the deprivation estimates to those of the WLS model.

^vi^Note that the clusters in Figures 3, 4, 5 include the cluster core and neighbouring postcode sectors. A queen contiguity criterion is used to define neighbours, i.e. postcode sectors with shared borders or corners. Because postcode sectors with population sizes below 1,000 were excluded from the sample, a lot of sectors no longer shared borders with nearby postcodes (in addition to real islands). To avoid disconnected postcode sectors, we converted the postcode sector geographic file to Thiessen polygons. This queen contiguity weights matrix is used for the spatial lag analysis throughout the paper.

^vii^Note that the percentages do not add up to 100% since spatial outliers, i.e. postcodes with high values surrounded by low values, and vice versa, are not included in the table.

^viii^Although the robust WLS model for West Scotland has the highest R2 value (0.87), it cannot be directly compared to the other R2 values, which are computed for both the West and rest of Scotland as spatial regimes.

^ix^To create the indicator variables, the deprivation values were sorted in ascending order and grouped into three equal intervals of 280 postcode sectors. The group with the lowest values represents the low deprivation indicator, the group with the highest values the high deprivation indicator and the group with the middle values is excluded as the base.

## Supplementary Material

Additional file 1 Table S1 - Distribution of Variables Click here for file

Additional file 2 Table S2 - Spatial Model Results Click here for file
