# Supplementary material for: Does context matter for the relationship between deprivation and all-cause mortality? The West vs. the rest of Scotland
Source: Int J Health Geogr. 2011 May 12;10:33. doi: 10.1186/1476-072X-10-33 (PMC3103414; doi:10.1186/1476-072X-10-33)
Supplement: Additional file 1 — Table S1 - Distribution of Variables [file 1476-072X-10-33-S1.PDF]

	Scotland		Rest		West	
	N = 840		N = 461		N = 379	
	Mean	Std. Dev.	Mean	Std. Dev.	Mean	Std. Dev.
SMR	99.46	35.96	87.54	27.68	113.96	39.42
SMR Males	100.02	40.25	87.36	30.50	115.42	45.07
SMR Females	98.66	35.58	87.75	29.84	111.93	37.47
Deprivation (Carstairs)	0.99	3.49	-0.05	2.60	2.26	3.99
% Male	48.25	1.69	48.67	1.67	47.75	1.57
% Female	51.75	1.69	51.33	1.67	52.25	1.57
% Age 0-4	5.35	1.09	5.29	1.10	5.43	1.08
% Age 5-14	12.32	2.64	12.21	2.73	12.46	2.51
% Age 15-24	12.28	5.56	12.04	6.62	12.57	3.90
% Age 25-34	13.54	4.30	13.35	4.58	13.77	3.94
% Age 35-54	29.11	3.41	29.36	3.75	28.82	2.92
% Age 55-64	11.15	2.20	11.26	2.36	11.00	1.98
% Age 65-74	8.97	2.31	9.00	2.30	8.93	2.31
% Age 75+	7.28	2.56	7.50	2.52	7.01	2.59
Urban Indicator	0.30	0.46	0.25	0.44	0.35	0.48
Annual Rainfall (inches)	91.96	31.61	77.42	28.44	109.66	25.71
Mean Temperature (degrees Celsius)	7.94	0.60	7.76	0.66	8.15	0.42
