# Supplementary material for: Does context matter for the relationship between deprivation and all-cause mortality? The West vs. the rest of Scotland
Source: Int J Health Geogr. 2011 May 12;10:33. doi: 10.1186/1476-072X-10-33 (PMC3103414; doi:10.1186/1476-072X-10-33)
Supplement: Additional file 2 — Table S2 - Spatial Model Results [file 1476-072X-10-33-S2.PDF]

Table 2.1

	Robust WLS			Robust OLS				Robust Spatial Lag (2SLS/IV)			
	DV: SMRs Total										
	Model 1	Model 2		Model 3	Model 4			Model 5	Model 6		
	Scotland	West	Rest	Scotland	West	Rest	Spatial Chow	Scotland	West	Rest	Spatial Chow
Intercept (1)	155.22 **	204.58 **	147.32 *	119.50 *	197.95 *	103.07	0.73	129.47 **	232.43 **	95.21	1.99
(2)	3.34	2.83	2.34	2.12	2.26	1.52		2.55	3.06	1.57	
Deprivation	8.65 ***	9.37 ***	8.14 ***	9.27 ***	9.78 ***	8.34 ***	3.69	8.33 ***	8.86 ***	8.13 ***	1.37
	30.67	23.36	18.42	22.97	17.92	16.26		23.21	19.61	17.39	
Low											
High											
% Male	0.42	1.41	0.46	0.83	2.41	0.17	1.79	0.38	1.83	0.22	1.35
	0.67	1.34	0.57	1.02	1.79	0.17		0.54	1.67	0.25	
% Age 0-4	1.23	0.84	-0.38	-0.67	0.14	-1.36	0.39	-0.57	0.27	-1.37	0.58
	1.15	0.54	-0.28	-0.57	0.07	-0.96		-0.51	0.16	-1.03	
% Age 5-14	-2.89 ***	-5.05 ***	-1.03	-3.05 ***	-5.68 ***	-0.61	11.39 ***	-2.12 **	-4.78 ***	-0.47	12.28 ***
	-5.89	-8.64	-1.03	-3.93	-5.34	-0.57		-3.19	-5.57	-0.53	
% Age 15-24	-1.55 **	-2.07 **	-1.12	-1.28 *	-2.70 **	-0.37	4.38 *	-1.12 *	-2.82 ***	-0.38	5.63 *
	-3.35	-3.05	-1.52	-2.33	-3.11	-0.53		-2.16	-3.54	-0.58	
% Age 25-34	-0.75	-1.90 **	-0.25	-0.82	-2.41 **	0.30	4.60 *	-0.49	-2.24 **	0.32	5.13 *
	-1.48	-2.76	-0.31	-1.36	-2.51	0.36		-0.86	-2.62	0.43	
% Age 35-54	-0.68	-0.52	-0.7449331	0.16	-0.62	0.15	0.31	-0.25	-1.63	0.10	1.93
	-1.34	-0.59	-0.98	0.25	-0.55	0.19		-0.41	-1.59	0.14	
% Age 55-64	-1.15	-2.24 **	-0.67	-1.63 *	-2.74 **	-0.29	3.30	-1.22	-2.88 **	-0.27	4.49 *
	-1.78	-2.76	-0.82	-2.40	-2.60	-0.35		-1.92	-3.02	-0.35	
% Age 65-74	-3.49 ***	-3.76 **	-2.83 *	-2.75 **	-4.78 **	-1.40	2.71	-2.55 **	-4.97 **	-1.38	3.62
	-3.99	-3.27	-1.95	-2.66	-3.24	-0.98		-2.61	-3.62	-1.06	
Urban Indicator	5.39 **	0.35	6.90 **	2.86	2.52	3.96 *	0.21	1.33	0.56	3.15	0.89
	2.79	0.17	2.88	1.81	1.04	2.03		0.96	0.27	1.74	
Annual Rainfall	0.07 *	-0.01	-0.02	0.05 *	0.00	-0.05	1.13	0.03	0.03	-0.06	0.05
	2.45	-0.37	-0.56	2.44	0.10	-1.29		1.45	0.91	-1.49	
Mean Temp.	3.65 **	0.64	0.46	3.90 ***	0.65	0.75	0.00	2.79 *	1.18	0.65	3.02
	2.96	0.32	0.32	3.35	0.32	0.48		2.51	0.60	0.46	
* < 0.05	** < 0.01	***< 0.001									
Spatial Lag of Y								0.11 **	0.07		
								2.82	1.74		
N	840	379	461	840	379	461		840	379	461	
R^2/Pseudo R^2	0.79	0.87	0.74	0.77	0.78			0.74	0.76		
Spatial Chow Test					40.82 ***				36.31 ***		

(1) beta, (2) t-value (OLS) or z-value (spatial 2SLS): Same for all variables

Table 2.2

	Robust Spatial Lag (2SLS/IV)												
	DV: SMRs Total			DV: SMRs Male			DV: SMRs Female			DV: SMRs Total			
	Model 7			Model 8			Model 9			Model 10			
	West	Rest	Spatial Chow	West	Rest	Spatial Chow	West	Rest	Spatial Chow	West	Southeast	North	Sp. Chow
Intercept (1)	245.80 *	12.39	3.56	176.65	36.79	1.12	345.63 **	1.02	6.59 **	245.06 *	83.52	22.41	2.50
(2)	2.38	0.18		1.59	0.52		3.13	0.01		2.37	1.10	0.21	
Deprivation													
Low	-5.86 *	-5.16 **	0.04	-7.10 *	-5.19 *	0.24	-2.86	-4.74 *	0.21	-5.73 *	-4.15	-7.31 **	0.73
	-2.07	-2.67		-2.21	-2.35		-0.86	-1.99		-2.03	-1.60	-2.73	
High	26.77 ***	21.50 ***	1.49	31.00 *	25.08 ***	1.43	23.62 ***	16.50 ***	1.80	27.22 ***	21.11 ***	21.03 ***	2.20
	8.15	7.47		7.95	7.84		5.72	4.78		8.33	6.32	4.69	
% Male	0.56	-0.85	0.64							0.51	-2.79 **	-0.65	3.97
	0.40	-0.79								0.36	-2.71	-0.46	
% Age 0-4	0.42	0.40	0.00	2.79	-0.02	0.72	-1.75	1.14	0.66	0.52	-0.55	0.22	0.11
	0.18	0.26		0.99	-0.01		-0.60	0.57		0.22	-0.24	0.10	
% Age 5-14	0.56	3.74 ***	4.01 *	-0.28	2.75 **	2.83	1.30	3.27 **	1.08	0.55	4.52 ***	2.46	5.67
	0.42	4.25		-0.18	2.91		0.85	2.90		0.42	4.35	1.79	
% Age 15-24	-2.69	0.76	7.28 **	-1.35	0.10	1.00	-3.93 ***	0.24	9.70 **	-2.63 **	0.65	0.84	7.14 *
	-2.56 **	1.02		-1.10	0.13		-3.69	0.29		-2.51	0.69	0.78	
% Age 25-34	-0.44	2.41 **	3.80 *	-0.57	1.68 *	2.19	-0.10	2.04 **	1.42	-0.33	3.01 ***	1.97	4.99
	-0.35	3.04		-0.44	2.09		-0.06	2.47		-0.27	3.48	1.47	
% Age 35-54	-5.68 ***	-1.51	7.45 **	-4.46 ***	-1.81 *	2.58	-6.13 ***	-2.17 *	5.21 *	-5.61 ***	-1.17	-1.94	7.15 *
	-4.45	-1.80		-3.18	-2.10		-4.26	-2.25		-4.40	-0.94	-1.69	
% Age 55-64	-1.29	0.63	1.32	-1.23	-1.18	0.00	-1.23	2.00 *	2.95	-1.35	1.91	1.07	3.32
	-0.96	0.65		-0.87	-1.18		-0.78	1.95		-1.00	1.57	0.80	
% Age 65-74	-4.30 *	2.30	8.01 **	-2.75	2.14	3.29	-5.65 **	1.11	6.09 **	-4.13 *	1.45	1.33	5.77
	-2.32	1.60		-1.26	1.34		-2.67	0.62		-2.23	0.79	0.62	
Urban Indicator	5.84 *	1.11	1.53	5.59	0.87	1.35	4.26	3.44	0.04	5.93 *	1.10	-1.50	2.38
	1.95	0.46		1.71	0.35		1.28	1.37		1.98	0.37	-0.34	
Annual Rainfall	0.07	0.01	0.88	0.09 *	0.02	1.20	0.03	-0.01	0.24	0.07	0.03	0.03	0.47
	1.86	0.31		2.24	0.39		0.54	-0.15		1.88	0.44	0.65	
Mean Temp.	2.45	0.55	0.42	5.51 *	-0.11	2.86	-1.56	0.27	0.23	2.52	-0.26	3.51	1.69
	1.00	0.34		1.95	-0.06		-0.51	0.12		1.03	-0.12	1.66	
* < 0.05	** < 0.01	***< 0.001											
Spatial Lag of Y	0.41 ***			0.41 ***			0.43 ***			0.38 ***			
	6.47			6.09			5.34			6.39			
N	379	461		379	461		379	461		379	229	232	
R^2/Pseudo R^2	0.63			0.60			0.50			0.64			
Spatial Chow	18.31			16.49			16.46			52.36 **			

(1) beta, (2) t-value (OLS) or z-value (spatial 2SLS): Same for all variables
